# A sex-inducing pheromone triggers cell cycle arrest and mate attraction in the diatom *Seminavis robusta*

**DOI:** 10.1038/srep19252

**Published:** 2016-01-20

**Authors:** Sara Moeys, Johannes Frenkel, Christine Lembke, Jeroen T. F. Gillard, Valerie Devos, Koen Van den Berge, Barbara Bouillon, Marie J. J. Huysman, Sam De Decker, Julia Scharf, Atle Bones, Tore Brembu, Per Winge, Koen Sabbe, Marnik Vuylsteke, Lieven Clement, Lieven De Veylder, Georg Pohnert, Wim Vyverman

**Affiliations:** 1 Protistology and Aquatic Ecology, Department of Biology, Ghent University, 9000 Ghent, Belgium; 2 Department of Plant Systems Biology, VIB, B–9052 Ghent, Belgium; 3 Department of Plant Biotechnology and Bioinformatics, Ghent University, B–9052 Ghent, Belgium; 4 Institute for Inorganic and Analytical Chemistry, Bioorganic Analytics, Friedrich Schiller University, 07743 Jena, Germany; 5 Department of Biology, California State University, 9001 Stockdale Highway, Bakersfield, CA 93311, USA; 6 Department of Applied Mathematics, Computer Science and Statistics, Ghent University, 9000 Ghent, Belgium; 7 Department of Biology, Norwegian University of Science and Technology, 7491 Trondheim, Norway

## Abstract

Although sexual reproduction is believed to play a major role in the high diversification rates and species richness of diatoms, a mechanistic understanding of diatom life cycle control is virtually lacking. Diatom sexual signalling is controlled by a complex, yet largely unknown, pheromone system. Here, a sex-inducing pheromone (SIP^+^) of the benthic pennate diatom *Seminavis robusta* was identified by comparative metabolomics, subsequently purified, and physicochemically characterized. Transcriptome analysis revealed that SIP^+^ triggers the switch from mitosis-to-meiosis in the opposing mating type, coupled with the transcriptional induction of proline biosynthesis genes, and the release of the proline-derived attraction pheromone. The induction of cell cycle arrest by a pheromone, chemically distinct from the one used to attract the opposite mating type, highlights the existence of a sophisticated mechanism to increase chances of mate finding, while keeping the metabolic losses associated with the release of an attraction pheromone to a minimum.

Diatoms are a key ecological and extremely diverse group of microalgae^1^ that contribute to about 20 percent of the global carbon fixation^2^. Their unique diplontic life cycle, involving a size-dependent transition from mitotically dividing cells to cells capable of sexual reproduction^3^,^4^, is a defining feature of diatom biology^5^. This size-dependent transition is connected to their cell wall structure, which consists of two overlapping halves or thecae. After cell division, each daughter cell inherits one theca and synthesizes a new one while still enclosed in the cell wall of the mother cell, leading to a decrease in mean cell size of the population with every cell division (Fig 1). Restoration of cell size typically occurs by zygote (auxospore) expansion following sexual reproduction. Cells that fail to reproduce sexually will continue to divide mitotically until they reach a critical minimal size, upon which they die. Strikingly, cells are only capable of sexual reproduction once a certain cell size (the sexual size threshold or SST) is reached^3^.

Major clades within the diatoms have evolved a diversity of sexual strategies, including homothallic reproduction within one clone and heterothallic reproduction with each clone belonging to one of two mating types (MT^+^ or MT^−^)^4^,^5^. Although it has been long suspected that diatoms employ chemical cues to facilitate mate-finding and sexual reproduction^4^,^6^,^7^,^8^,^9^, the first molecular structure of a diatom pheromone was only recently revealed for the model species *Seminavis robusta*^10^. In this species, a two-step signalling system has been identified, in which mating-compatible cells below the SST produce sex-inducing pheromones (in the following abbreviated as SIP) that trigger the production of the proline-derived diketopiperazine (in the following abbreviated as diproline)^10^ (Fig. 1). Diproline then functions as an attraction pheromone to direct MT^+^ cells towards MT^−^ cells, followed by pairing of the cells and meiosis. Compared to other microalgae^11^,^12^,^13^, this pheromone is active at relatively high concentrations. Here, we used physiological, metabolomic, and transcriptomic methods to provide a deep molecular insight into the two-step signalling system of *S. robusta.* Specifically, we isolated and physicochemically characterized the sex-inducing pheromone produced by MT^+^ cells (SIP^+^) that initiates the biosynthesis and secretion of diproline by MT^−^ cells. This isolated SIP^+^ was then used in RNA sequencing experiments to study the pheromone signalling on the transcriptional level. We also verified the existence of a chemically distinct SIP that is produced by MT^−^ cells (SIP^−^) and found that both chemically distinct SIPs, besides sexualizing cells, reciprocally arrest the cell cycle at the G1 phase.

## Results

### Cells below the SST produce cytostatic pheromones

**Figure 1:**
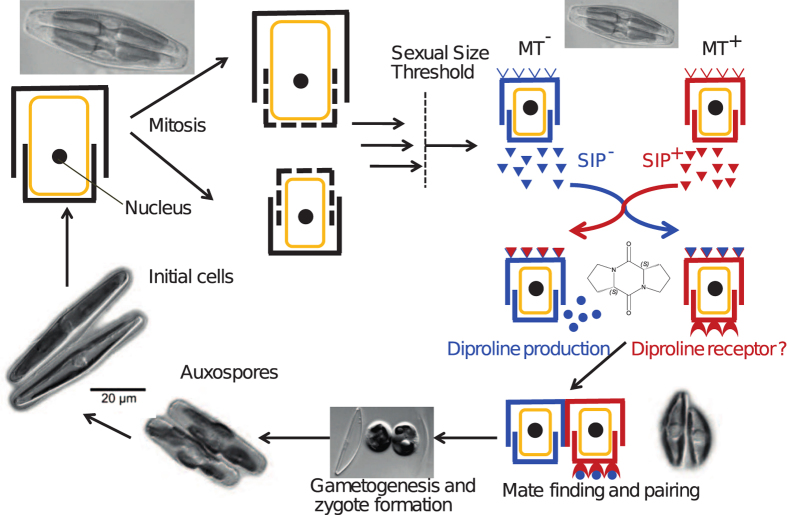
The life cycle of *Seminavis robusta,* modified after Frenkel, *et al.*^5^ with microscopic images from Chepurnov, *et al*.^24^ and Chepurnov, *et al*.^9^. During the vegetative phase, average cell size decreases. Once below a certain cell size, called the sexual size threshold (SST), diatoms become capable of sexual reproduction. When passing this SST, both MT^+^ and MT^−^ cells start the production of sex-inducing pheromones, SIP^+^ and SIP^−^ respectively. Under the influence of these pheromones, MT^−^ secretes the attraction pheromone diproline and MT^+^ probably expresses a diproline receptor. Diproline signalling then leads to mate finding and pair formation, after which gametes and zygotes are formed. The specialized zygotes, called auxospores, elongate to initial cell size.

**Figure 2:**
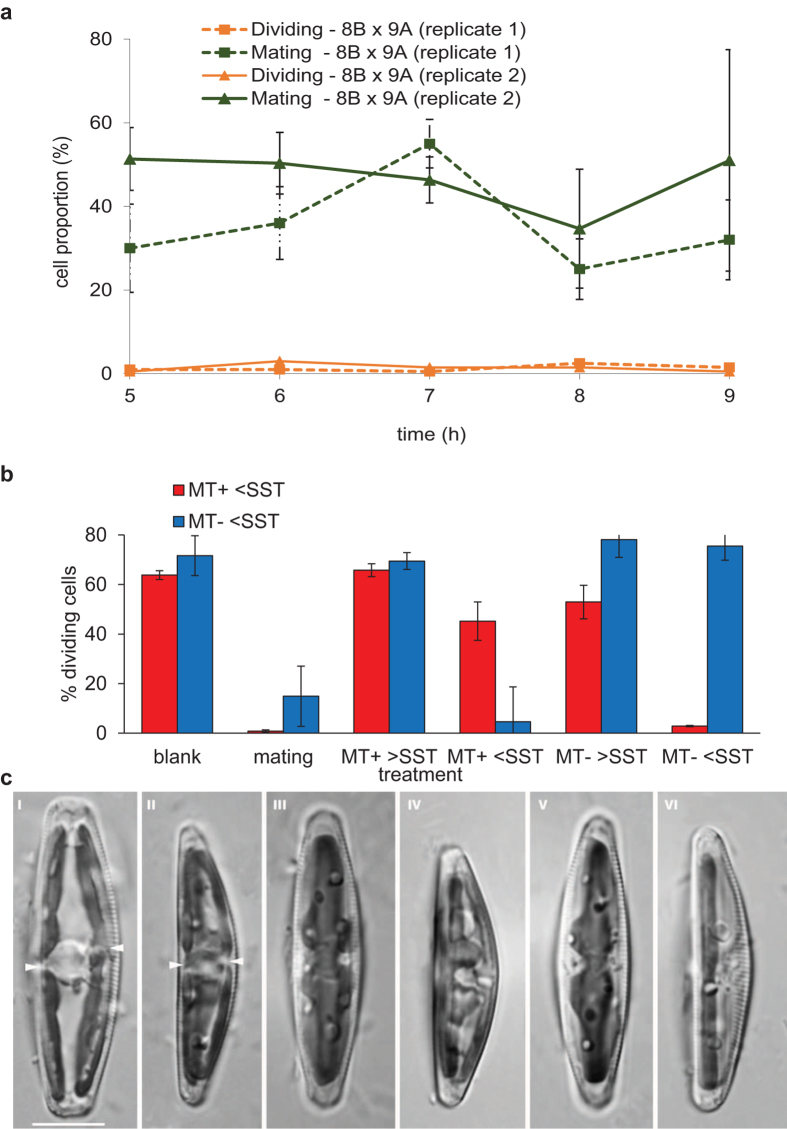
Cells below the SST produce a cytostatic pheromone. (**a**) The proportion of dividing and mating cells was determined by counting the different cell types under an inverted microscope in two mixed cultures of MT^+^ (8B) × MT^−^ (9A) strains after dark-synchronization. A minimum of 200 instances was evaluated for every time point. Error bars are ± s.e. of 3 replicate estimations. (**b**) Means ± s.e. (n = 3) of proportions of mitotically dividing MT^+^ (red) or MT^−^ (blue) cells below the SST, 9 h after illumination, depending on the administration of fresh medium (blank), medium from a mating culture or medium from the opposite mating partner (above or below the SST). (**c**) Light microscopy images of MT^−^ (85A) cells treated for 8 h with control medium (i–ii), with filtered medium of MT^+^ (85B) cells below the SST (iii–iv), or with purified SIP^+^ (v–vi). Cells are shown in the girdle (i, iii, v) or valve (ii, iv, vi) view. Arrowheads in i and ii point to constricting plastids. Scale bar =  10 μm.

When two strains of opposite mating types with cell sizes below the SST are mixed, mating pairs are formed. In these pairs, gamete formation is initiated (Fig. 1), implying a switch from mitotic cell cycle progression to meiosis. Surprisingly, while only about half of the cells engage in mating pair formation, virtually none of the cells in the co-culture continued mitotic cell division (Fig. 2a). Replacing the culture medium of MT^+^ or MT^−^ cells with a size below the SST by filtered culture medium of a mating culture (containing all excreted metabolites, but no cells) resulted for both in the induction of cell cycle arrest. Experiments with combinations of opposite mating types showed that two specific signals are involved, as only filtered medium from the MT^+^ culture induced cell cycle arrest in the MT^−^ culture and vice versa (Fig. 2b). Cells with a size above the SST did not respond to the same treatments, nor were they able to induce cell cycle arrest in other cultures (Supplementary Fig. S1). Microscopic observations showed that growth-arrested cells had undivided chloroplasts at the girdle (Fig. 2c and Supplementary Fig. S2). During vegetative division, chloroplasts divide and translocate from the girdle to the valves during the S/G2 phase^14^. The location and morphology of the chloroplasts of growth-arrested cells are thus indicative for the G_1_ phase. Together, these results suggest that cells below the SST secrete mating type-specific pheromones with the ability to arrest the cell cycle of the cells of the compatible mating type in the G_1_ phase.

### Metabolomics-assisted identification of SIP^+^

**Figure 3:**
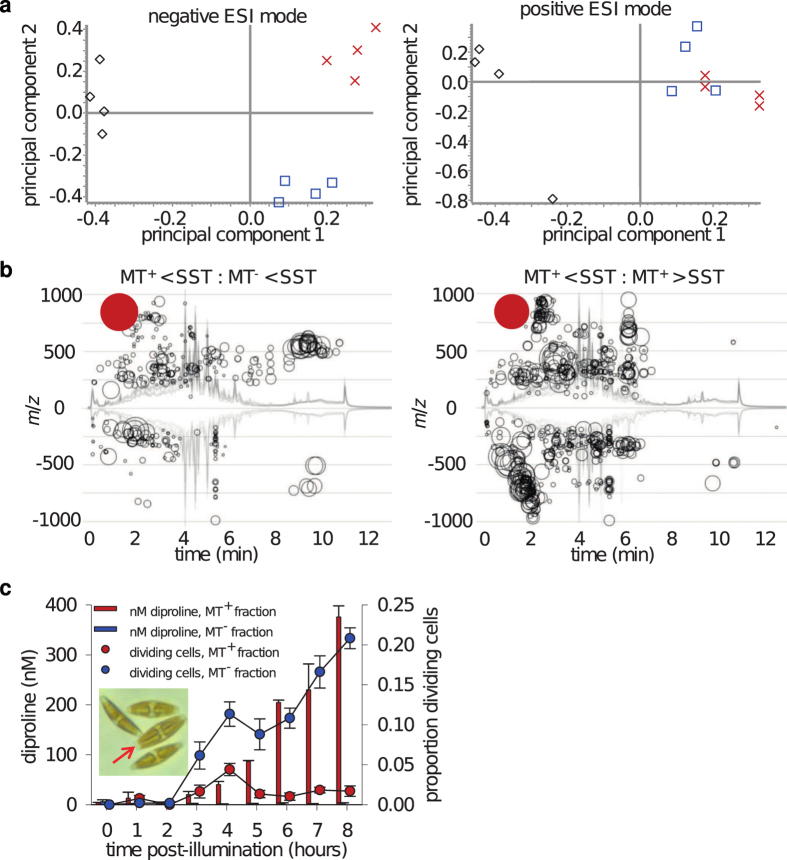
Metabolomics-Assisted Identification of SIP^+^. (**a**) Principal component analysis of Liquid-chromatography (LC)-mass spectrometry data in positive and negative ESI mode from extracts (n = 4) of MT^+^ cells above the SST (black), MT^+^ cells below the SST (red) and MT^−^ cells below the SST (blue). (**b**) Screening of up- (positive Y-axis) and down- (negative Y-axis) regulated metabolites in MT^+^ cells below the SST compared with MT^−^ cells below the SST (left) and MT^+^ cells above the SST (right) in the chromatograms acquired in negative ESI mode (n = 4). The radius of the circle shows the relative degree of regulation of the signal with the indicated mass at the respective retention time in the chromatogram. The red circle corresponds to 600x (left) or 320x (right) upregulation in comparison of the treatments and indicates a signal with *m*/*z* = 842. (**c**) Means ± s.e. (n = 3) of diproline concentration in the medium and dividing MT^−^ cells below the SST treated with purified fractions of MT^+^ below the SST containing SIP^+^ (red) and fractions of equally purified fractions of MT^−^ below the SST (blue), acting as control. The red arrow shows a dividing cell.

In addition to inducing cell cycle arrest, medium of MT^+^ cells below the SST activates the production of the attraction pheromone diproline in MT^−^ cells^10^. To assess if the signal leading to cell cycle arrest is identical to the compound inducing diproline production, we aimed to purify the putative pheromone(s). Based on the above results, we reasoned that cell size-dependent production of a signal molecule would be reflected by changes in the exo-metabolome (i.e. all metabolites released into the medium)^15^. We therefore performed a comparative exo-metabolomic study of MT^+^ versus MT^−^ cells below the SST and of MT^+^ cells below versus MT^+^ cells above the SST to identify unique compounds in the pheromone-emitting cultures. For this approach, suitable extraction procedures for SIP^+^ were developed using bioassays to confirm that the extract could induce diproline production in MT^−^ and therefore contained the pheromone. Positive bioassay results were observed when the medium was extracted using reversed-phase solid-phase extraction (RP-SPE) cartridges. Similar to the filtered culture medium of MT^+^ cells below the SST, these partially purified extracts induced diproline production in MT^−^, indicating that SIP^+^ is extractable with the applied technique. Subsequently, the extracts were submitted to reversed-phase ultra-performance liquid chromatography/mass spectrometry (RP-UPLC/MS) and measured in positive and negative electrospray-ionization (ESI) mode. The resulting chromatograms were highly complex, covering diatom metabolites and medium ingredients, as well as contaminants from solvents and plasticizers. In order to identify metabolites specific for MT^+^ cells below the SST, data were resolved with principal component analysis. The replicates of the exo-metabolomes measured in negative mode grouped according to their respective cell size and mating type; data recorded in positive ionization mode revealed fewer differences (Fig. 3a). Chromatograms measured in negative mode were screened for metabolites that varied in their concentration using XCMS online, a platform for processing untargeted metabolomics data^16^. A metabolite having a pseudomolecular ion of [M-H]^−^ = 842 was identified with the most pronounced upregulation in MT^+^ cells below the SST compared with MT^−^ cells below the SST (Fig. 3b). It also displayed the second highest upregulation in MT^+^ cells below the SST compared with MT^+^ cells above the SST (Fig. 3b). This metabolite showed a rather high polarity eluting from a C18 RP-UPLC column at a solvent composition of 15% acetonitrile in water. A UPLC fraction containing this purified metabolite indeed induced cell cycle arrest and the production of the attraction pheromone diproline in MT^−^ (Fig. 3c), thereby supporting the compound as a candidate for SIP^+^.

### Further purification and partial characterization confirmed the identity of SIP^+^

**Figure 4:**
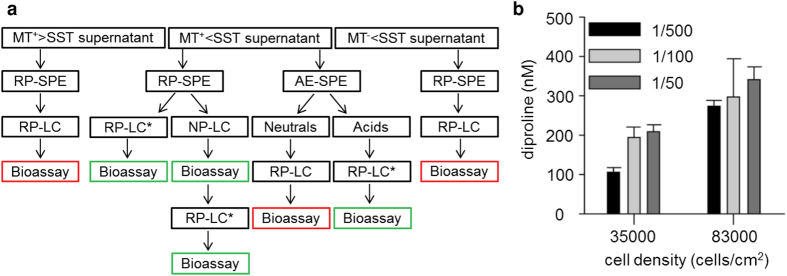
Further purification of SIP^+^. (**a**) Orthogonal purification of this metabolite through a combination of reversed-phase solid-phase extraction (RP-SPE), anion exchange (AE)-SPE, RP-liquid chromatography (LC) and normal-phase (NP)-LC. The algae supernatant, SPE-extract or LC-fraction were applied in diproline-induction assays. A positive result was only observed when the signal with *m/z* = 842 was detected (green), whereas there was no bioactivity when the metabolite could not be found (red). *RP-LC was accomplished with two different gradients. (**b**) Means ± s.e. (n = 3) of diproline concentration in the medium of MT^−^ cells below the SST after treatment with different amounts of SIP^+^.

In order to rule out the possibility that co-eluting metabolites in the fraction containing the SIP^+^ candidate cause the attraction pheromone induction in MT^−^ cells, the metabolite was further purified, guided by a targeted UPLC-MS analysis (Fig. 4a). First, to achieve a higher selectivity during the extraction process, strong anion-exchange SPE (AE-SPE) was used instead of RP-SPE to enrich acidic metabolites from the culture medium. Indeed, the extract, and not the flow through, induced diproline production in MT^−^ cultures. Accordingly, only the AE-SPE extract contained the SIP^+^ candidate with a pseudomolecular mass of [M-H]^−^ = 842. To exclude potential artefacts during purification, a flatter gradient was developed for elution; again, only the fraction containing the SIP^+^ candidate was bioactive (Fig. 4a), confirming that the metabolite with [M-H]^−^ = 842 is the diproline-inducing SIP^+^. For further characterization of the metabolites, high-resolution (HR) ESI-MS/MS experiments were conducted. Analysis of the isotope pattern corroborated the presence of at least one sulphur atom in the molecular formula and fragmentation supported the presence of a sulphate functionality in the metabolite (Supplementary Fig. S3). Due to the very low amounts of released SIP^+^, further attempts for structure elucidation after scale-up proved to be unsuccessful. Whereas purification of one litre of MT^−^ culture was sufficient to extract 100 μg diproline, not even 20 litres of MT^+^ medium could yield any weighable amounts of SIP^+^. Considering the low amount of SIP^+^ in the medium, one must conclude that its activity is remarkably high. The medium of a culture of 35,000 cells/cm^2^ could be diluted 500-fold before a dose-dependent reduction in diproline production in induced MT^+^ cells could be observed (Fig. 4b).

### SIP^+^ is responsible for the mitosis-to-meiosis switch in MT^−^ cells

**Figure 5:**
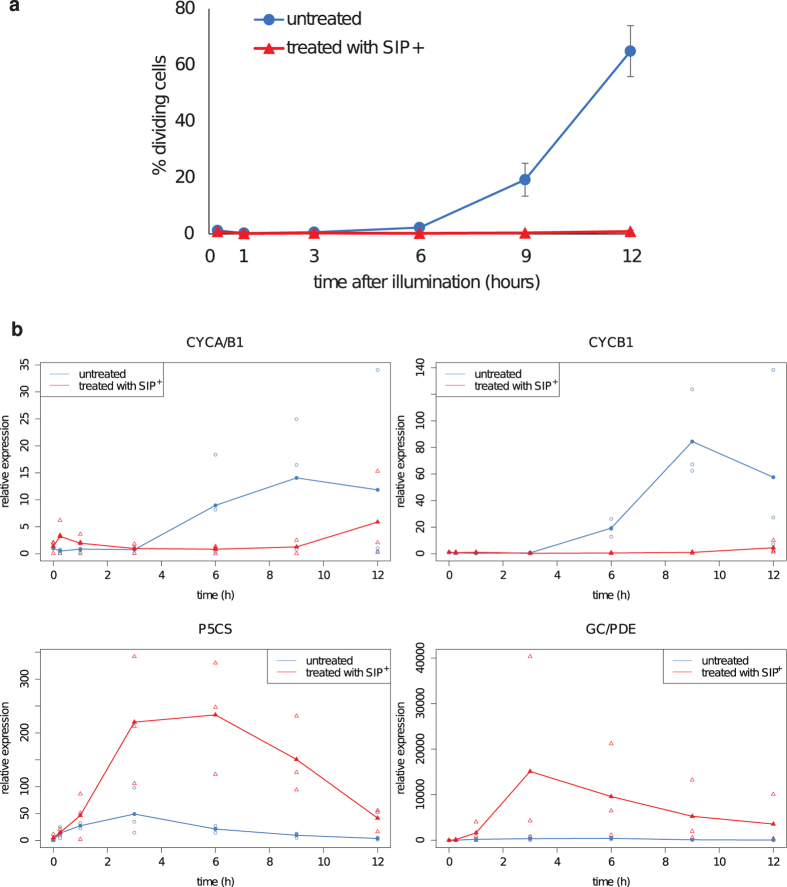
Transcriptional effects of SIP^+^ on MT^−^ cells below the SST determined by qRT-PCR. (**a**) Mean ± s.e. (n = 3) of the percentage of dividing MT^−^ cells after treatment with SIP^ + ^(red) or untreated (blue). (**b**) Mean (n = 3) relative expression of *CYCA/B1, CYCB1, P5CS* and *GC/PDE* in SIP^ + ^-treated (red) and untreated (blue) MT^−^ cultures. Biological replicates (n = 3) are shown as separate data points. Relative expression values are normalized to two constitutively expressed genes.

The cell cycle arrest induced by SIP^+^ was verified by expression profiling of cell cycle marker genes. Cultures of MT^−^ cells below the SST were grown for three days in a 12-h:12-h light:dark regime, after which the cell cycle was synchronized by extending the dark period for twelve additional hours. After this prolonged dark period, the majority of the cells were arrested in the G_1_ phase of the cell cycle^14^. Half of the cultures were treated with SIP^+^ before illumination. The expression levels of two mitosis markers were measured by real-time quantitative PCR (RT-qPCR) to investigate the regulation of cell cycle arrest. The *CYCB1* and *CYCA/B1* markers were selected because they are highly expressed during mitosis in the diatom *Phaeodactylum tricornutum*^17^. *CYCB1* and *CYCA/B1* were indeed expressed during mitosis in untreated *S. robusta* cells. In contrast, their expression was repressed when the cells were treated with SIP^+^, which corroborates the observed cell cycle arrest (Fig. 5a,b).

**Figure 6:**
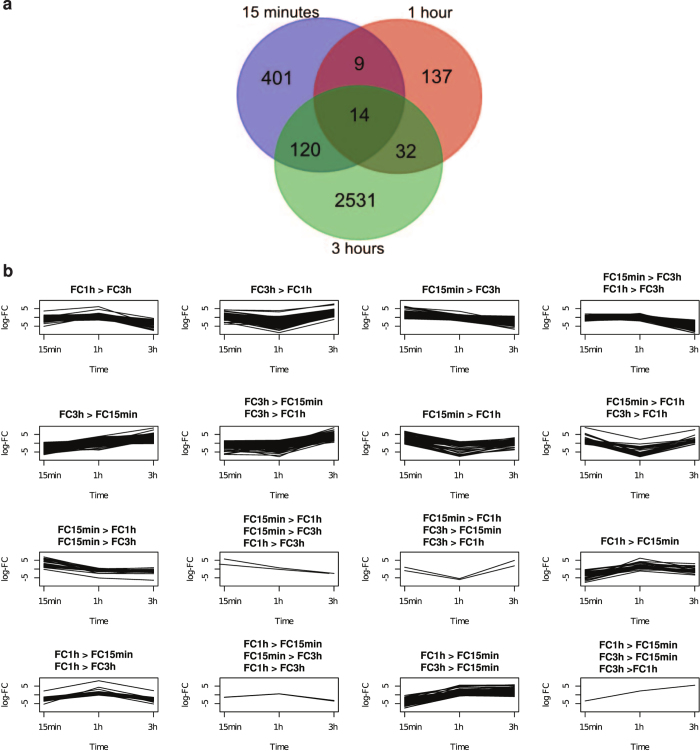
RNA-seq experiment of SIP^+^-treated and untreated MT^−^ cultures. (**a**) Venn diagram showing the number genes that are differentially expressed between SIP^+^-treated and untreated cultures at 15 min, 1 h and 3 h after illumination. (**b**) Transcripts for which the fold change (treated versus untreated cells) changed over time, divided in classes based on the direction of the difference in logFC between the different time points. FC15 min = log fold change at 15 min, FC1 h = log fold change at 1 h, FC3 h = log fold change at 3 h.

In order to obtain a more global view on the transcriptional changes induced by SIP^+^, an RNA-seq experiment was conducted. Therefore, purified SIP^+^ from the RP-UPLC was added to MT^−^ cultures, 15 min before light onset after a prolonged dark period. MT^−^ cultures without extract addition constituted the control conditions. Samples were taken after 15 min, 1 h and 3 h of illumination. An additional, non-treated MT^−^ culture was harvested before illumination. RNA extracted from these samples was sequenced and resulting reads were assembled in a transcriptome containing 50,231 transcripts (Supplementary Table S1). Comparing gene expression data of treated and untreated samples for each time point showed 3,461 unique transcripts being differentially expressed in at least one time point (Supplementary Table S2). The majority of these differentially expressed transcripts was observed at 3 h (2,697 transcripts), while 192 and 544 transcripts were differentially expressed at 1 h and 15 min, respectively (Fig. 6a). Furthermore, a significant disparity in fold change (FC) was observed for 289, 1,308 and 1,263 transcripts in the 1 h versus 15 min, 3 h versus 15 min and 3 h versus 1 h comparisons, respectively. These results were used to classify the differentially expressed transcripts according to the significant FC patterns (Fig. 6b).

Amongst the differentially expressed genes after treatment with SIP^+^, four meiosis-related genes were identified, all being significantly higher expressed in treated cultures compared with untreated ones after 3 h of illumination (Supplementary Fig. S4). Although some show already small fold changes at 15 min and 1 h, the fold change is most outspoken at 3 h (ranging from 4.7 to 13.4). MRE11 and RAD50 are part of the Mre11-Rad50-Nbs1 complex that is involved in repairing DNA double-strand breaks and homologous recombination during meiosis^18^,^19^. MSH4 is a mismatch repair protein that functions specifically in meiosis^20^. MCM8 is involved in meiotic recombination^21^,^22^. Although not much is known about the exact role of these four proteins in diatoms, there are indications that they are indeed involved in meiosis^23^. Along with the observed cell cycle arrest, these data indicate that SIP^+^ sensing by MT^−^ cells below the SST results in a switch from mitosis-related genes to meiosis-related genes, although meiosis itself will only start when mating pairs have been formed and gametogenesis can start^24^.

### The glutamate-to-proline pathway is transcriptionally upregulated in response to SIP^+^ treatment

Four hours after illumination and conditioning with SIP^+^ extracts, diproline was detected in the medium of the dark-synchronized MT^−^ cultures (Fig. 3c). Thus, we expected the genes responsible for diproline biosynthesis to be upregulated in the transcriptome after administration of SIP^+^. Correspondingly, Δ1-pyrroline-5-carboxylate synthetase (*P5CS;* asmbl_5165 Table S2) was time-dependently upregulated in treated cultures (Fig. 6b, subset FC3h > FC15min, FC3h > FC1h), with a strong increase in expression at 3 h. This enzyme has both γ-glutamyl kinase and glutamic-γ-semialdehyde dehydrogenase activity and mediates the first two steps in the conversion of glutamate to proline^25^. Consequently, this enzyme could catalyse the first steps in diproline biosynthesis. The second enzyme of the pathway, Δ1-pyrroline-5-carboxylate reductase (P5C; asmbl_35385 Table S2), was also significantly higher expressed in treated cultures compared with untreated ones 3 h after illumination. Because diproline is detected in treated MT^−^ cultures from 4 up to 8 h after illumination (Fig. 3c), the expression of *P5CS* was monitored with a higher resolution over 12 h by RT-qPCR, showing that *P5CS* expression is already slightly induced 1 h after illumination and peaks at 3–6 h in the treated cultures (Fig. 5b). This time profile corresponded with the timing of mate pairing in *S. robusta* (5–10 h after illumination)^10^.

### The expression of a bifunctional guanylyl cyclase/phosphodiesterase increases upon treatment with SIP^+^

The only transcript for which the FC in expression in treated cultures at each time point was statistically higher than at the previous time point (class FC3h > FC1h, FC3h > FC15min, FC1h > FC15min) (Fig. 6b), encodes a putative bifunctional protein containing an adenylyl/guanylyl cyclase domain and a cyclic nucleotide phosphodiesterase (PDE) domain (asmbl_30773 Table S2). These domains are responsible for cyclic nucleotide (cAMP or cGMP) synthesis and breakdown, respectively, pointing to the potential involvement of one of these secondary messengers in pheromone signalling in *S. robusta*. The catalytic core of the cyclase domain was aligned to that of several adenylyl and guanylyl cyclases that were previously used to model the catalytic mechanism of nucleotide cyclases (NCs)^26^. Of the guanine binding residues, all but two are conserved and except for the pyrophosphate-binding site, all catalytic sites are conserved (Supplementary Fig. S5). Richter *et al.*^27^ identified eight amino acids that are conserved in cAMP-specific PDEs but not in cGMP-specific PDEs. These amino acids are not conserved in the PDE domain of this *S. robusta* protein (Supplementary Fig. S5), indicating that it probably has cGMP-hydrolyzing capacity.

Guanylyl cyclases can be either transmembrane or soluble proteins. Prediction of the transmembrane topology of the bifunctional guanylyl cyclase (GC)/PDE^28^ indicated two predicted transmembrane domains N-terminal from the two catalytic domains, suggesting that this probably is a transmembrane guanylyl cyclase. The transcription of the *GC/PDE* gene was followed during a longer time course by RT-qPCR, which indicated that its expression peaks at three hours after supplementing with SIP^+^ (Fig. 5b).

## Discussion

Using a comparative metabolomics approach, a pheromone, called SIP^+^, that is exclusively produced by MT^+^ cells below the SST was purified. SIP^+^ triggers both cell cycle arrest and diproline production in MT^−^ cells. Despite the minute amount of produced pheromone, metabolomics-assisted identification of a SIP^+^ candidate, in combination with orthogonal separation protocols could unambiguously identify a metabolite with a pseudomolecular ion of [M-H]^−^ = 842 in negative ESI-MS. HR-MS and fragmentation analysis identified this compound as a sulfated metabolite. Interestingly, *S. robusta* uses the cyclic dipeptide diproline as an attraction pheromone, but the mass spectral data suggest that SIP^+^ is structurally unrelated. SIP^+^ is more polar, potentially sulphated and of significantly higher molecular weight compared to diproline. In general, the pheromone chemistry of (micro)algae is highly diverse and covers a vast range of polarity and molecular weight^5^. Our identification of SIP^+^ indicates that diatoms invoke most likely multiple metabolic pathways in producing chemically distinct metabolites as pheromones, even within a single species. Strikingly, SIP^+^ appears to possess extreme high activity. Likewise, some known pheromones in aquatic systems are active at very low concentrations. Lurlenic acid, which is produced by MT^−^ cells of *Chlamydomonas allensworthii* to attract MT^+^ cells, is active at picomolar concentrations^11^, whereas in *Volvox* species pheromones are known to induce gametogenesis at even lower concentrations (from 10^−12^ to even 10^−16^)^12^,^29^.

As in yeast, mammals and plants, commitment to meiosis in *S. robusta* is made during the G_1_ phase, before the onset of the S phase^30^, as evident from the chloroplast behaviour^14^,^24^. Similarly, in the centric diatom *Thalassiosira weissflogii*, cells can only be induced to undergo spermatogenesis when they are in the early G_1_ phase^31^. In accordance with these observations, we showed that SIP^+^ induces cell cycle arrest in the G_1_ phase in *S. robusta*. Likewise, MT^−^ cells induce cell cycle arrest in MT^+^ cells via the production of a so far unidentified SIP^−^ pheromone. This reciprocal pheromone signalling is reminiscent of the system known in yeast where pheromones induce cell cycle arrest in G_1_-phase preceding mating^32^. Whereas in yeast, cell cycle arrest precedes directional cell growth leading to fusion, cell cycle arrest in *S. robusta* accompanies the secretion of the attraction pheromone diproline. This two-step signalling thus effectively increases chances of finding a mating partner within the right time frame and physiological conditions, which appears particularly relevant in diffusion-controlled environments, such as biofilms. It allows signalling compounds to operate on a local scale and develop concentration gradients on spatial scales relevant to those covered by cells gliding through a structurally complex matrix.

Transcriptome analysis revealed that SIP^+^ induced a significant modulation of gene expression patterns. About 6% of the transcripts were differentially expressed between controls and SIP^+^-treated cells, providing a first insight into the molecular pathways involved. Notably, the genes Δ1-pyrroline-5-carboxylate synthetase and Δ1-pyrroline-5-carboxylate reductase were upregulated in response to SIP^+^. These genes encode two enzymes from the pathway responsible for the conversion of glutamate to proline, suggesting that the attraction pheromone diproline is synthesized from an increasing intracellular pool of proline. However, candidate enzyme(s) implicated in the last steps (proline to the cyclic dipeptide (CDP) diproline) could not yet be identified. CDPs are widespread in nature and are predominantly synthesized by microorganisms. They are thought to be involved in cell-to-cell communication, for instance in bacterial quorum sensing or in bacteria-plant interactions^33^,^34^. Two distinct protein families are known to be involved in CDP biosynthesis, namely non-ribosomal peptide synthetases (NRPSs) and CDP synthetases (CDPSs) (31). While CDPSs are highly specific for substrates with only the canonical amino acids, NRPSs can use a wider range of substrates. Several putative NRPS homologues were expressed in the *S. robusta* transcriptome, but none of them was upregulated upon treatment with SIP^+^. Nevertheless, it is possible that these genes are constitutively expressed and that their activity is regulated at the posttranscriptional level or by substrate availability, which would be in accordance with the results described above. Further, although no CDPS homologue could be identified using PSI-blast, it cannot be excluded that an enzyme with a similar function is present, because sequence similarity between CDPS orthologues is only 19–27%^35^.

Interestingly, a bifunctional guanylyl cyclase/phosphodiesterase (GC/PDE) was found for which the expression difference between treated and untreated cells strongly increased over time. cGMP is a secondary messenger synthesized from GTP by GCs and is broken down to GMP by PDEs. GCs can be soluble or membrane-bound. In animals, soluble GCs are activated by nitric oxide, while membrane-bound GCs are activated by ligands that bind to their extracellular domain^36^. The SIP^+^-responsive GC/PDE is predicted to have two transmembrane domains, and both the putative GC and PDE catalytic domains are positioned at the inside of the cell. We hypothesize that this enzyme is activated by a ligand (most likely SIP^+^) that binds to its extracellular domain. Mammalian membrane-bound GCs have one transmembrane domain with the catalytic domain on the inside of the cell and a ligand-binding domain on the outside, but other conformations have been found in other eukaryotes^37^. The main targets of cGMP are cGMP-dependent protein kinase, cGMP-gated ion channels, and proteins containing a GAF-domain. cGMP is known to regulate many processes in mammals, including smooth muscle relaxation and phototransduction^38^,^39^. cGMP signalling is often involved in motility, for example in sperm chemotaxis in the sea urchin *Arbacia punctulata* or myosin II regulation in *Dictyostelium*^40^,^41^. Up till now, the function of cGMP signalling in diatoms remains unclear. The fact that the GC and PDE domains are part of a fusion protein could indicate a tight regulation of cGMP levels. To our knowledge, this combination of domains is only found in diatoms. We hypothesize that SIP^+^ is sensed by either the extracellular domain of GC/PDE or by an activator of this protein, leading to cGMP synthesis, which can then activate the downstream signalling cascade, probably resulting in cell cycle arrest and diproline production.

In conclusion, the data presented here combined with the hypothesized existence of a yet unidentified SIP^−^ that is produced by MT^−^ cells and putatively induces or activates the diproline receptor in MT^+^, indicate that *S. robusta* has developed a rather complex reciprocal signalling system that controls the initial steps of sexual reproduction (Fig. 1). The control of diproline production and perception by two sex-inducing pheromones seems to be a high investment compared with a continuous secretion of diproline by *S. robusta* once cell size drops below the SST. However, SIP^+^ and putatively SIP^−^ are secreted in much lower concentrations than diproline. These pheromones only transmit the information of the presence of the algae and not their location. Only when the opposite sex perceives the presence of its mating partner, it starts the potentially more expensive production of the N-rich diproline in elevated concentrations as well as its receptor. Consequently, metabolic losses of resources are minimized since their release into the environment is limited to a very short time frame.

## Methods

### Strains and culture conditions

*Seminavis robusta* strains 8B and 85A (MT^+^) and 9A and 85B (MT^−^) were obtained from the diatom culture collection of the Belgian Coordinated Collection of Micro-organisms (BCCM/DCG, http://bccm.belspo.be, accession numbers DCG 0097, DCG 0105, DCG 0096 and DCG 0107). Cultures were either grown in F/2 medium made with autoclaved, filtered natural sea water collected from the North Sea and Guillard’s F/2 solution (Sigma-Aldrich)^42^ or in artificial sea water^43^.

### Metabolomic screening by RP-UPLC/MS analysis

Three strains of *S. robusta* (cell size: MT^+^ cells below the SST = 22.4 μm, MT^−^ cells below the SST = 23.5 μm, MT^+^ cells above the SST = 51.9 μm; four replicates each) were grown in 200 ml medium until the culture reached a density of around 100,000 cells/cm^2^ in 185-cm^2^ flasks. The cultures were filtered five hours after illumination and the filtrate was extracted on 200 mg HLB-SPE cartridges (Oasis^®^, Waters, Eschborn, Germany), following the manufacturer’s instructions. The eluate was evaporated to dryness under a stream of nitrogen and dissolved in 100 μl bidistilled water. After centrifugation, 20 μl of the supernatant was analysed with an UPLC system coupled to a time-of-flight mass spectrometer with electrospray ionization (ESI-MS) (Acquity UPLC^®^/Q-ToF micro Waters, Manchester, UK) with an Acquity UPLC BEH C_18_ column (2.1 × 50 mm, 1.7 μm, Waters). The composition of the mobile phase was set to 100% A (0.1% formic acid and 2% acetonitrile in water) and ramped to 100% B (0.1% formic acid in acetonitrile) in a linear gradient within 10 min with a flow of 0.6 ml/min. The solvent composition was held at 100% B for 1 min, returned to 100% A in 0.1 min and was kept at 100% A for 2.4 min. The scanned mass range was between 50 and 1000 *m/z* with a scan rate of 0.9 scans sec^−1^ and an inter-scan delay of 0.1 sec. The sample cone was set to 25 V, the cone gas flow was held at 20 l N_2_ h^−1^ and the desolvation gas flow at 700 l N_2_ h^−1^. Principal component analysis was done using an established method by means of the Software MarkerLynx (Waters, Milford, USA)^44^. The screening for up-regulated metabolites was performed with XCMS Online (Scripps Center for Metabolomics, La Jolla, USA)^16^. SIP^+^ was fractionated at 1.4 min (15% B) and added to a dark-synchronized^14^ MT^−^ culture (cell size: 23 μm, three replicates per fraction), which was grown in 20 ml medium in 25-cm^2^ flasks and had a cell density of around 50,000 cells/cm^2^. The diproline concentration in the medium was checked eight hours after illumination following an established method by extracting the medium with HLB-SPE cartridges (Oasis^®^, Waters, Eschborn, Germany) and investigating the extract using gas chromatography coupled to mass spectrometry on an ISQ Trace GC-Ultra GC-MS system (Thermo Fisher, Dreieich, Germany)^10^.

### Purification of SIP^+^

Besides RP-SPE with HLB-SPE cartridges, alternative extraction of SIP^+^ from the culture medium was done with AE-SPE, using mixed mode MAX SPE-cartridges (Oasis^®^, Waters, Eschborn, Germany) following the manufacturer instructions. For optimization of the separation of SIP^+^ in RP-UPLC/MS, all factors in the UPLC-MS analysis were kept unchanged compared to the above protocol, except the solvent gradient, which was ramped from 0% to 10% B in 8 min, and fractions containing SIP^+^ were gathered at 4.6 min (6% B). For NP-HPLC/MS, the analytical column was changed to a SeQuant® ZIC®-HILIC HPLC column (150 × 2.1 mm, 5 μm, Merck). The composition of the mobile phase was kept at 100% B (90% acetonitrile with 5 mM NH_4_Ac) for 1 min and ramped to 80% A (0.1% formic acid and 2% acetonitrile in water) within 5.5 min, returned to 100% B within 0.6 min and was held for 2.9 min. The pheromone was fractionated at 3.9 min (42% A). All gathered fractions were tested for their diproline-inducing capability in MT^−^ as mentioned above.

### Mass spectrometric characterization of SIP^+^

For structural characterization of SIP^+^, 10 μl of MT^+^ medium extract was analysed by HPLC (Dionex UltiMate® 3000, Thermo Fisher Scientific, Dreieich, Germany), coupled to an ESI-Orbitrap MS (Q-Exactive Plus, Thermo Fisher Scientific, Dreieich, Germany).

Liquid chromatography was done with a Kinetex® C18 column (2.1 × 50 mm, 1.7 μm, Phenomenex, Torrance, CA, USA). The composition of the mobile phase was set to 100% A (0.1% formic acid and 2% acetonitrile in water) for 0.2 min and ramped to 100% B (0.1% formic acid in acetonitrile) in a linear gradient within 7.8 min. The solvent composition was held at 100% B for 1 min, returned to 100% A in 0.1 min and held at 100% A for 0.9 min. The flow rate was adjusted to 0.4 ml min^−1^.

Ionization was performed with a spray voltage of 3.3 kV and a capillary temperature of 360 °C. The sheath gas flow rate was kept at 60 arbitrary units. Nitrogen was used as desolvation gas. The scanned mass range was between *m/z* 100 and 1,500, at *m/*Δ*m* 280,000 resolving power for high-resolution mass spectrometry and at *m/*Δ*m* 17,500 resolving power for MS^2^ in negative ion mode. MS^2^ was done by fragmenting the precursor pseudomolecular ion [M-H]^−^ = 842.20 *m/z* with a collision energy of 20 eV.

### Cell cycle arrest

Triplicate MT^+^ and MT^−^ cultures with cell sizes above and below the SST were grown separately in 12-well tissue culture plates and G1-phase synchronized using the dark-arrest method described by Gillard *et al.*^14^. Then, their supernatant medium was replaced with filtered medium from either MT^+^ cultures (with a size above or below the SST), MT^−^ cultures (with a size above or below the SST), a mixed mating culture (MT^+^ × MT^−^) or fresh culture medium. The cultures were then kept in the dark for two more hours and cell cycle progression was assessed at 9 h after illumination by counting the number of dividing cells (minimum of 200 instances were evaluated).

For testing the cell cycle arresting capability of SIP^+^, MT^+^ and MT^−^ cells with a size below the SST were cultured in 200 ml medium in 185-cm^2^ tissue culture flasks and the medium was extracted with HLB-SPE as described above. The extracts were submitted in sequence to RP-UPLC/MS, fractionated at 4.6 min (6% B) and evaporated under a stream of nitrogen. After re-dissolving in one ml of water, both extracts were equally distributed to each 27 dark-synchronized MT^−^ cultures grown in 20 ml medium in 25-cm^2^ flasks with a cell density of around 15,000 cells/cm^2^. After re-illumination, three replicates were processed per hour by counting the amount of dividing cells and measuring the extracellular concentration of diproline as described above.

### RNA-seq on SIP^+^-treated MT^−^ cultures

Cultures of strain 85B (MT^−^) with an average cell size below the SST were grown at 18 °C in a 12-h:12-h light:dark regime with cool white fluorescent lamps at approximately 80 μmol photons m^−2^ s^−1^. Before sampling, the dark period was extended with 12 h to synchronize the cells in the G_1_ phase. A first control sample was taken in darkness (T0). Then, SIP^+^ was added to half of the cultures in darkness. After 15 min, the light was turned on and 15 min later both treated and untreated cultures were harvested by filtration on Versapor filters (3-μm pore size, 25-mm diameter, PALL). The filters were rinsed with 1 ml PBS and put in liquid N_2_. This was repeated 1 h and 3 h after illumination. Sampling was carried out in triplicate. The protocol used for RNA extraction was based on the method developed by Apt, Clendennen, Powers and Grossman^45^ with some modifications. The frozen filters were put in extraction buffer (100 mM Tris-HCl pH 7.5, 2% CTAB, 1.5 M NaCl, 50 mM EDTA, 10% β-mercaptoethanol) and sharp carbid beads were added. The samples were shaken in a bead mill (Retsch) at room temperature for 30 min. 100 μl of 10X Chelex-100 was added and the sample was incubated for 15 min at 56 °C, then one volume of chloroform:isoamyl alcohol (24:1, v/v) was added and the samples were shaken for 25 min at low speed. After centrifugation, the upper phase was transferred to a new tube and mixed with 0.3 volumes of absolute ethanol to precipitate the polysaccharides, and extracted with 1 volume of chloroform. After centrifugation, the upper phase was transferred to a fresh tube and RNA was precipitated overnight at −20 °C by addition of 0.25 volumes of 12 M LiCl and β-mercaptoethanol to 1% final concentration. After centrifugation, the pellet was washed with 70% ethanol, air-dried and suspended in RNase-free water for DNase treatment by an RNase-free DNase I (Turbo DNase, Ambion) according to the manufacturer’s instructions. An extraction was then carried out by adding phenol-chloroform (1:1, V/V). After centrifugation, the upper phase was transferred to a fresh tube, and extracted with 1 volume of chloroform:isoamyl alcohol (24:1, V/V) and centrifuged again. The upper phase was precipitated with 0.3 M NaAc pH 5.5 and 75% ice cold ethanol by incubating overnight at 20 °C. After centrifugation, the pellet was washed with 70% ethanol and air-dried. The pellet was resuspended in an appropriate volume of RNase-free water. RNA quality was evaluated by spectrophotometry (Nanodrop) and Bioanalyzer (Agilent Technologies).

Sequencing libraries were prepared using Illumina TruSeq Stranded mRNA kit. All libraries were analysed in a 2 × 150 bp run on 2 lanes of a flowcell of the Illumina HiSeq 2500 at the VIB Nucleomics Core ( www.nucleomics.be). Every library was sequenced twice, as all libraries were pooled together in every lane. After adaptor trimming with CLC Assembly Cell 4.2.0 (CLC Inc, Aarhus, Denmark), all paired reads were mapped to an in-house draft genome of *S. robusta* using GSNAP (GMAP-GSNAP version 2013-06-27)^46^ with default settings. Based on this mapping, a genome-guided trinity approach was used to assemble the transcriptome, using Trinity r20131110^47^ and PASA r20130907^48^. All reads were mapped to this transcriptome using GSNAP^46^ after which all unmapped reads were extracted and de novo assembled using Trinity^47^ with default settings. This *de novo* assembly was joined together with the genome-guided transcriptome assembly. CD_HIT 4.6.1 (cd-hit-est)^49^,^50^ was used to optimize the assembly with the following settings: -c 0.9 -n 8 -t 1. Finally, all transcripts shorter than 500 bp were discarded. For functional annotation, the final transcriptome was loaded into TRAPID (reference database: Plaza 2.5)^51^,^52^. For every library, the reads were mapped to the final transcriptome using GSNAP^46^ with default settings and mapped reads per transcript per library were counted. Transcripts with low overall counts (threshold of at least 1 cpm in at least six samples) have been removed from the analysis because they have little power for detecting differential expression. Hence, filtering these transcripts leads to a negligible information loss and results in a gain of statistical power^53^. Upon filtering, the sequencing libraries were normalized using TMM normalization^54^. Transcript-wise RNA-seq counts were analysed using a negative binomial model with a factor with 7 levels, one for each treatment (Control, SIP^+^) × time (T0, T1, T2, T3) combination (C0, C1, C2, C3, SIP^+^ 1, SIP^+^ 2, SIP^+^ 3). The control taken in darkness (C0) was used as the reference level. Technical replicates have been pooled to avoid underestimation of the dispersion parameter. Both differential expression (DE) between SIP^+^-treated samples and untreated samples within each time point as well as DE changes over time have been assessed on a log scale using appropriate contrasts (DE1 = SIP^+^ 1 – C1, DE2 = SIP^+^ 2 – C2, DE3 = SIP^+^ 3 – C3, DE3-DE1, DE2-DE1, DE3-DE2). A hierarchical two-stage approach has been used for assessing statistical significance, which controls the overall false discovery rate (FDR) at the transcript level^55^. In the first stage, the overall null hypothesis that no differential expression occurred at none of the time points (DE1 = DE2 = DE3 = 0) has been assessed. Next, the six contrasts of interest have been tested for all genes that passed the screening stage. An advanced, within-gene, multiple testing correction that accounts for the relatedness of different hypotheses (contrasts) has been adopted in the second stage^56^. Hence, the overall FDR has efficiently been controlled at a 5% level, while maintaining significant power for testing the hypotheses of interest. All analyses were conducted with the R/BioConductor package edgeR (version 3.8.5)^57^ in R version 3.1.2 and likelihood ratio tests were used for assessing all hypotheses of interest^58^. RNA-Seq data have been submitted to ArrayExpress ( www.ebi.ac.uk/arrayexpress), with accession number E-MTAB-4056.

### qRT-PCR on SIP^+^-treated MT^−^ cultures

Samples were taken as described for the RNA-seq on SIP^+^-treated MT^−^ cultures just before illumination and at 15 min, 1 h, 3 h, 6 h, 9 h and 12 h after illumination. Progression through the cell cycle was monitored by assessing the percentage of dividing cells using an inverted microscope. The total RNA was extracted using the RNeasy Plant Mini Kit (Qiagen). About 200 ml of culture (±5 × 10^6^ cells) was scraped from the bottom of a tissue culture flask (Cellstar, 175 cm^2^ growth surface, Greiner Bio-One) and filtered on a Versapor filter (3-μm pore size, 25-mm diameter, PALL). Filters were frozen in liquid nitrogen. RLT buffer (1 ml), β-mercaptoethanol (10 μl) and silicon carbide beads (1.0 mm, BioSpec) were added to the filter. Cells were disrupted by beating on a bead mill (Retsch, 3 × 1 min). The remaining steps for the RNA extraction were done according to the manufacturer’s instructions. cDNA was prepared using the iScript cDNA Synthesis Kit (Bio-Rad) as recommended by the manufacturer.

Fifteen ng of cDNA was used as a template for each qRT-PCR reaction. Samples were amplified in triplicate on the Lightcycler 480 platform with the Lightcycler 480 SYBR Green I Master mix (Roche) in the presence of 0.5 μM gene-specific primers (primer sequences in Supplementary Table S3). The cycling conditions were: 10 min of polymerase activation at 95 °C and 45 cycles at 95 °C for 10 s, 58 °C for 15 s, 72 °C for 15 s. Afterwards, amplicon dissociation curves were recorded by heating from 65 °C to 97 °C with a ramp rate of 2.5 °C. Data were analysed in the qbase + software package (Biogazelle) with the ΔCt relative quantification method with two stably expressed normalization genes, previously selected based on cDNA-AFLP data (Devos V., unpublished data).

## Additional Information

**How to cite this article**: Moeys, S. *et al.* A sex-inducing pheromone triggers cell cycle arrest and mate attraction in the diatom *Seminavis robusta. Sci. Rep.* **6**, 19252; doi: 10.1038/srep19252 (2016).

## Supplementary Material

Supplementary Information

Supplementary Table S2
