# Supplementary material for: A sex-inducing pheromone triggers cell cycle arrest and mate attraction in the diatom Seminavis robusta
Source: Sci Rep. 2016 Jan 20;6:19252. doi: 10.1038/srep19252 (PMC4726125; doi:10.1038/srep19252)
Supplement: Supplementary Information [file srep19252-s1.pdf]

Supplementary Information

A sex-inducing pheromone triggers cell cycle arrest and mate attraction in the diatom *Seminavis robusta*

Sara Moeys[1,2,3†], Johannes Frenkel[4†], Christine Lembke[4†], Jeroen T. F. Gillard [1,5], Valerie Devos[1,2,3], Koen Van den Berge[6], Barbara Bouillon[1,2,3], Marie J. J. Huysman[2,3], Sam Dedecker[1], Julia Scharf[4], Atle Bones[7], Tore Brembu[7], Per Winge[7], Koen Sabbe[1], Marnik Vuylsteke[2,3], Lieven Clement[6], Lieven De Veylder[2,3], Georg Pohnert[4], Wim Vyverman[1]

[1]Protistology and Aquatic Ecology, Department of Biology, Ghent University, 9000 Ghent, Belgium; [2]Department of Plant Systems Biology, VIB, B–9052 Ghent, Belgium; [3]Department of Plant Biotechnology and Bioinformatics, Ghent University, B–9052 Ghent, Belgium; [4]Institute for Inorganic and Analytical Chemistry, Bioorganic Analytics, Friedrich Schiller University, 07743 Jena, Germany; [5]Department of Biology, California State University, 9001 Stockdale Highway, Bakersfield, CA 93311, USA; [6]Department of Applied Mathematics, Computer Science and Statistics, Ghent University, 9000 Ghent, Belgium; [7]Department of Biology, Norwegian University of Science and Technology, 7491 Trondheim, Norway

[†]These authors contributed equally to this work

Correspondence should be addressed to Lieven.DeVeylder@psb.vib-ugent.be, Georg.Pohnert@uni-jena.de or Wim.Vyverman@ugent.be

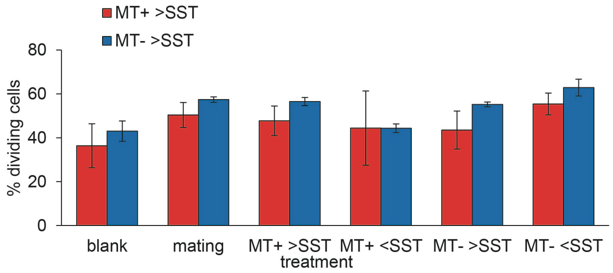

Supplementary Figure S1. The cytostatic pheromone has no effect on cells above the SST. Means ± s.e. (n = 3) of proportions of mitotically dividing MT^+ or MT^- cells above the SST, 9 h after illumination, depending on the administration of fresh medium (blank), medium from a mating culture or medium from the opposite mating partner (above or below the SST).

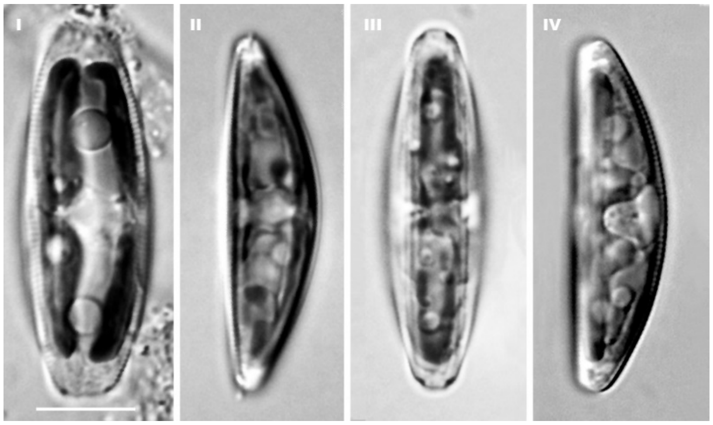

Supplementary Figure S2. Cytostatic effect of MT^- medium. Light microscopy images of MT^+ (85B) cells treated for 8 h with control medium (i-ii), or with filtered medium of MT^- (85A) cells below the SST (iii-iv). Cells are shown in the girdle (i, iii) or valve (ii, iv) view. Scale bar = 10 µm.

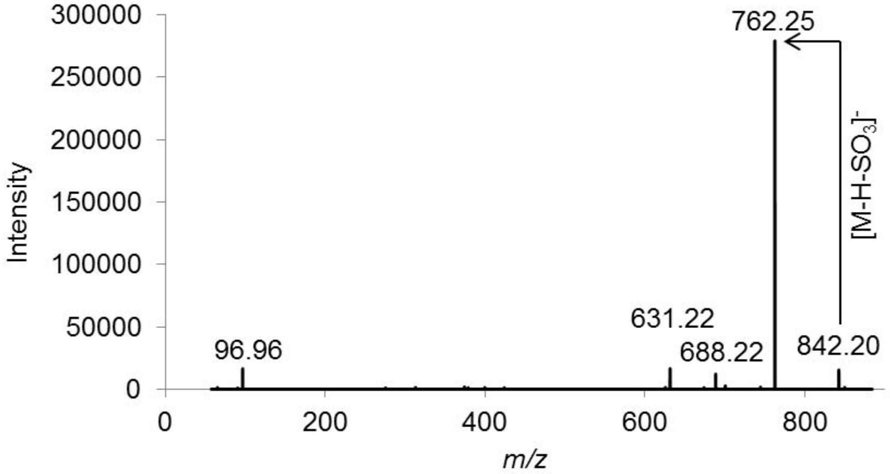

Supplementary Figure S3. Mass spectral analysis of SIP^+. The MS^2 spectrum of $[M\text{-}H]^-$ = 842 acquired by LC-ESI-Orbitrap mass spectrometry indicated a sulphated molecule.

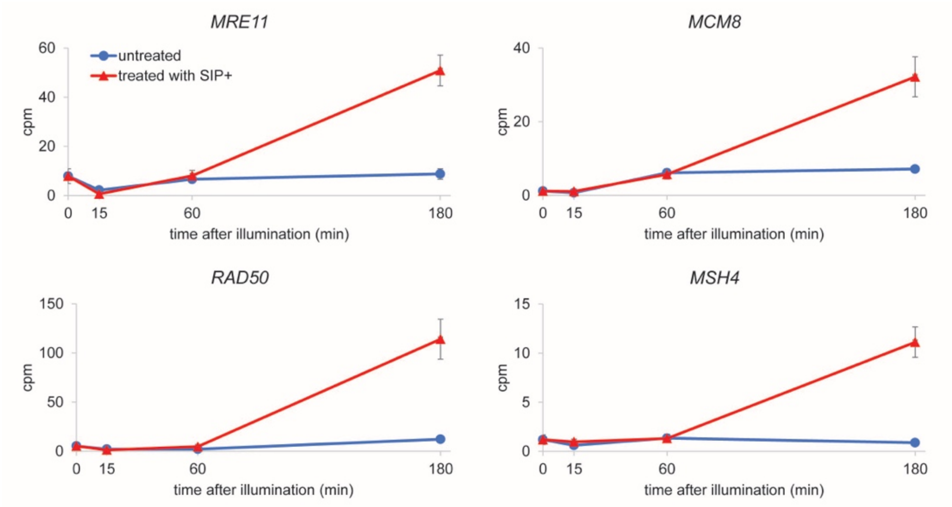

Supplementary Figure S4. Differential expression of meiosis-related genes after treatment with SIP^{+}. Mean ± s.e. (n=3) counts per million (cpm) for *MRE11, MCM8, RAD50 and MSH4* in untreated (blue) and treated (red) cultures.

a

```
BT_AC1_C1 291 RIFHKIYIQRHDNVSILFADIVGFTGLASQCT....AQELVKLLNELFGKFDELATE...NHCRRIKILGDCYYCVSGLT
BT_AC1_C2 857 PRNMDLYYQSYSQVGVMFASIPNFNDFYIELDGNNMGVECLRLLNEIIADFDELMDKDFYKDLEKIKTIGSTYMAAVGLA
BT_AC2_C2 872 LKNEELYHQSYDCVCVMFASIPDFKEFYTESDVNKEGLECLRLLNEIIADFDDLLSKPKFSGVEKIKTIGSTYMAATGLS
DD_ACG    382 ENGEDVVAERSNNACVFFLDIAGFTRFSSIHS....PEQVIQVLIKIFNSMDLLCAK...HGIEKIKTIGDAYMATCGIF
BT_sGCa   468 WQGHAVQAKRFGNVTMLFSDIVGFTAICSQCS....PLQVITMLNALYTRFDRQCGE...LDVYKVETIGDAYCVAGGLH
BT_sGCb   407 RHKRPVPAKRYDNVTILFSGIVGFNAFCSKHASGEGAMKIVNLLNDLYTRFDTLTDSRKNPFVYKVETVGDKYMTVSGLP
HS_rGC1   866 KTGTPVEPEYFEQVTLYFSDIVGFTTISAMSE....PIEVVDLLNDLYTLFDAIIGS...HDVYKVETIGDAYMVASGLP
SR_NC/PDE 582 NDSMKPIADLFPNTTVMFADISGFTAWSSTRD....PSQVFMLLQRIYGEFDEVARRRN...VFKVETIGDCYVACAGLP
                               * *    *                                         * * *

BT_AC1_C1 ...........QPKTDHAHCCVEMGLDMIDTITSVAEATE..VDLNMRVGLHTGRVLCGVL......GLRKWQYDVWSNDVTLANVME
BT_AC1_C2 ...PTAGTRAKKSISSHLSTLADFAIDMFDVLDEINYQSY..NDFVLRVGINVGPVVAGVI......GARRPQYDIWGNTVNVASRMD
BT_AC2_C2 AIPSQEHAQEPERQYMHIGTMVEFAYALVGKLDAINKHSF..NDFKLRVGINHGPVIAGVI......GAQKPQYDIWGNTVNVASRMD
DD_ACG    P........KCDDIRHNTYKMLGFAMDVLEFIPKEMSFHL...GLQVRVGIHCGPVISGVI.....SGYAKPHFDVWGDTVNVASRME
BT_sGCa   ...........KESDTHAVQIALMALKMMELSHEVVSPHG..EPIKMRIGLHSGSVFAGVV......GVKMPRYCLFGNNVTLANKFE
BT_sGCb   ...........EPCIHHARSICHLALDMMEIAGQVQVD.G..ESVQITIGIHTGEVVTGVI......GQRMPRYCLFGNTVNLTSRTE
HS_rGC1   ..........QRNGQRHAAEIANMSLDILSAVGTFRMRHMPEVPVRIRIGLHSGPCVAGVV......GLTMPRYCLFGDTVNTASRME
SR_NC/PDE ...........NKQPNHAAIMSRFAWECKSKLAVIL.KHLEVSLGPDTGDLSMRFGLHSGAVTAGVLRGDKARFQLFGDTVNTAARME
                                                                                  * ***   **   * *
```

b

```
HS_PDE4A      LENLNKWGLNIFCVSDYAGGRSLTCIMYMIFQERDLLKKFRIPVDTMVTYMLTLEDHYHADVAYHNSLHAADVLQSTHVLLATPA--------LD----------
HS_PDE7B      LSKVGMWDFDIFLFDRLTNGNSLVTLLCHLFNTHGLIHHFKLDMVTLHRFLVMVQEDYHSQNPYHNAVHAADVTQAMHCYLKEPK--------LA----------
HS_PDE8B1     LDNEESWDFNIFELEAITHKRPLVYLGLKVFSRFGVCEFLNCSETTLRAWFQVIEANYHSSNAYHNSTHAADVLHATAFFLGKER--------VK----------
BT_PDE5       TLKITDFSFSDFELS----DLETALCTIRMFTDLNLVQNFQMKHEVLCKWILSVKKNYRKNVAYHNWRHAFNTAQCMFAALKAGK--------IQ----------
HS_PDE6       SAELYEFRFSDFPLT----EHGLIKCGIRLFFEINVVEKFKVPVEVLTRWMYTVRKGYR-AVTYHNWRHGFNVGQTMFTLLMTGR--------LK----------
HS_PDE9       IEALRKPTFDVWLWE----PNEMLSCLEHMYHDLGLVRDFSINPVTLRRWLFCVHDNYR-NNPFHNFRHCFCVAQMMYSMVWLCS--------LQ----------
SR_NC/PDE     VEAIKLPDFDDNAVT---------------EEDLSFVHIPEVVVDQLRAHVTAIAESYQ-DNPFHNFEHACHVTMSTTKFLGRIASMEIDLPTLQGKDARAAVAS
                      :.                         . .        :   .  :   *:    :**  *   .       :            :

HS_PDE4A      AIHDVDHPGVSNQFLINTNSELALMYNDESVLENHHLAVGFKLL-QEDNCDI---FQNLS--------AVFTDLEILAALFAAKRQRQSLRKMVIDMVLATDMSK
HS_PDE7B      AAHDVDHPGVNQPFLIKTNHHLANLYQNMSVLENHHWRSTIGML-RESR--L---LAHLP--------SFLTPLDIMLGLLAAKEMTQDIEQQLGSLILATDINR
HS_PDE8B1     TVHDVDHPGRTNSFLCNAGSELAVLYNDTAVLESHHTALAFQLTVKDTKCNI---FKNID--------GSLDQLDEVAALIAARNHYRTLRQAIIDMVLATEMTK
BT_PDE5       LSHDLDHRGVNNSYIQRSEHPLAQLYC-HSIMEHHHFDQCLMIL-NSPGNQI---LSGLS--------KRLTDLEILALLIAAIEEYKTTLKIIKQAILATDLAL
HS_PDE6       FCHDIDHRGTNNLYQMKSTSPLARLHG-SSILERHHLEYSKTLL-QDESLNI---FQNLN--------KYYTDLEAFAMLAAAKRQFETVIHLFEVAIIATDLAL
HS_PDE9       ICHDLDHPGYNNTYQINARTELAVRYNDISPLENHHCAVAFQIL-AEPECNI---FSNIP--------EKFSQTDILILMTAAPDGFKQIRQGMITLILATDMAR
SR_NC/PDE     LIHDCDHAGVGNAQTIKENPKMGEKYQGKSVAEQNSLDIAWNLLMSERFAELRALLFANDHIHDYTHGILSDPLTLFGIVYSSKAEMARFRQVVVNIVLATDIFD
                ** ** *  :    .    :.  :   :  * .       :   .    :   :                    .  : ::        : .    ::**::

HS_PDE4A      HMTLLADLKTMVETKKVTSS-------GVLLLDNYSDRIQVLRNMVHCADLSNPTKPLELYRQWTDRIMAEFFQQGDRERERGME-ISPMCDKHT-AS
HS_PDE7B      QNEFLTRLKAHLHNK-------------DLRLEDAQDRHFMLQIALKCADICNPCRIWEMSKQWSERVCEEFYRQGELEQKFELE-ISPLCNQQK-DS
HS_PDE8B1     HFEHVNKFVNSINKPMAAEIEGSDCECNPAGKNFPENQILIKRMMIKCADVANPCRPLDLCIEWAGRISEEYFAQTDEEKRQGLPVVMPVFDRNT-CS
BT_PDE5       YIKRRGEFFELIMK------------NQFNLEDPHQKELFLAMLMTACDLSAITKPWPIQQRIAELVATEFFDQGDRERKELNIEPADLMNREKKNK
HS_PDE6       YFKKRTMFQKIVDACE---QMQTEEEAIKYVTVDPTKKEIIMAMMMTACDLSAITKPWEVQSQVALMVANEFWEQGDLERTVLQQQPIPMMDRNKRDE
HS_PDE9       HAEIMDSFKEKMENF--------------DYSNEEHMTLLKMILIKCCDISNEVRPMEVAEPWVDCLLEEYFMQSDREKSEGLP-VAPFMDRDK-VT
SR_NC/PDE     KE--LNGLRKGRWNKA--------FEESAEGSNSHLRATIVLEHIIQASDVSHTMQHWHIYRKWNRRLFLEMYSAYKQGRMGAD--PS--------SF
                     :                                .    : ..*:.   :   :       :  * :   .  :
```

Supplementary Figure S5. Conservation of the cyclase and phosphodiestase domains of GC/PDE. (A) Sequence alignment of the catalytic core of the following adenylyl/guanylyl cyclases: BT_ACI_C1 (NP_776654.1), BT_ACI_C2 (NP_776654.1), BT_ACII_C2 (NP_112269.1), DD_ACG (Q03101.2), BT_sGCα (NP_786972.1), BT_sGCβ (NP_777066.1), HS_rGC1 (AAI48422.1) and SR_NC/PDE (BT = *Bos Taurus*, DD = *Dictyostelium discoideum*, HS = *Homo sapiens*, SR = *Seminavis robusta*).The first four proteins are adenylyl cyclases, the next three are guanylyl cyclases. Adenine/guanine binding residues are indicated in yellow in SR_NC/PDE and catalytic sites are indicated in red, conversed residues are indicated with * (adapted from Liu et al. (1997)). (B) Sequence alignment of part of the following PDE proteins: three cAMP-specific PDEs (PDE4, PDE7 and PDE8), three cGMP-specific PDEs (PDE5, PDE6 and PDE9) and the bifunctional *S. robusta* GC/PDE. The eight amino acids conserved only in cAMP-hydrolyzing PDEs are indicated in red.

Table S1: General statistics on the *S. robusta* transcriptome

total number of transcripts	50231
total transcriptome length	71.10 Mbp
minimal transcript length	501 bp
maximal transcript length	17339 bp
average transcript length	1416 bp
N50	1730 bp

Table S2: See file: TableS2.xlsx

Table S3: Primers used in the RT-qPCR on CYCA/B1, CYCB1, P5CS and GC/PDE.

gene	forward primer	reverse primer
**V1**[1]	CAAGGCAAGAAGGATGGCAAG	GCGACAAGCAAATTAGCAAACC
**V4**[1]	AGGCTACCGTGGGACTTGG	GATCTGGACTGCGCTGGTTC
**CYCA/B1**	GCTTGGGTCGATGCAATACT	ATCCGAGAGGGTCAAGAGGT
**CYCB1**	GGGATGTAACAAACGCCAAT	CCTCTCATCCATGACGGACT
**P5CS**	AATCGAAAACGGGGTAGCTT	CCCTCGACAATCTCAACCAT
**GC/PDE**	TCCTTCCCAAGTTTTCATGC	CATGGTTTGGTTGCTTGTTG

[1] Normalization genes.
